# The Impact of Interpersonal Discrimination and Stress on Health and Performance for Early Career STEM Academicians

**DOI:** 10.3389/fpsyg.2016.00615

**Published:** 2016-04-28

**Authors:** Katharine R. O’Brien, Samuel T. McAbee, Michelle R. Hebl, John R. Rodgers

**Affiliations:** ^1^CUNA Mutual Group, Human Resources, Insights & Analytics, MadisonWI, USA; ^2^Department of Psychology, Illinois Institute of Technology, ChicagoIL, USA; ^3^Department of Psychology, Rice University, HoustonTX, USA; ^4^Department of Pathology and Immunology, Baylor College of Medicine, HoustonTX, USA

**Keywords:** interpersonal discrimination, incivility, physical and psychological health, STEM, academic productivity

## Abstract

The present study examines the consequences of perceived interpersonal discrimination on stress, health, and performance in a sample of 210 science, technology, engineering, and mathematics (STEM) academicians. Using a path model, we test the relation that perceived interpersonal discrimination has on stress and the relation of stress to physical health maladies and on current and future performance. In so doing, we assess the link between discrimination and decrements in performance over time. Additionally, we test supervisor social support as a moderator of the discrimination–stress relation. Findings support relations between perceived interpersonal discrimination and stress, which in turn relates to declines in physical health and performance outcomes. Moreover, supervisory support is shown to mitigate the influence of interpersonal discrimination on stress in STEM academicians.

## Introduction

Over the past several decades, increasing attention has been given to the representation of individuals of minority status in science, technology, engineering, and mathematics (STEM) fields (Halpern et al., 2007; Ceci et al., 2009, 2014; Halpern, 2014). Although, the minority gap is decreasing, women, in particular, continue to be underrepresented in math-intensive STEM fields (e.g., computer science, engineering; Su and Rounds, 2015; Wang and Degol, 2016). This underrepresentation of minorities in STEM fields arises from myriad factors that contribute to an individual’s decision to pursue and maintain a career in STEM, ranging from differences in vocational interests (e.g., Su et al., 2009; Su and Rounds, 2015) to various cultural and societal barriers faced by minority group members (e.g., Halpern et al., 2007). One pernicious barrier that minorities face is the experience of overt and subtle forms of discrimination (e.g., Wang and Degol, 2016). The experience of such discrimination likely contributes to the attrition of underrepresented minorities from STEM fields in later career stages (Riﬄe et al., 2013), known as the “leaky pipeline" (e.g., Metcalf, 2010). For those who remain in STEM, the experience of discrimination might also impact minority group members’ productivity and success in these fields. Research examining the impact of interpersonal discrimination on physical and psychological health and well-being, and, in particular, performance-based outcomes is critically needed (Jones et al., 2013). Understanding barriers that limit the retention and advancement of diverse STEM academicians is essential in stemming the outflow of such individuals from these fields. Thus, the present study investigates the impact of interpersonal discrimination and stress on health and performance outcomes, and the factors that mitigate the impact of interpersonal discrimination, in a sample of early-career STEM academicians.

### Discrimination in the Workplace

More than one-third of American adults report that they have experienced discrimination, bullying, harassment, or other forms of aggression due to their race, gender, appearance, or age at some point in their working career (Kessler et al., 1999). Research shows that experiencing workplace discrimination and the stress resulting from discrimination are related to a host of negative organizational and employee outcomes, such as lowered job satisfaction, reduced organizational commitment, reduced job performance, decreased helping behaviors, and increased turnover intentions (Ensher et al., 2001; Lim et al., 2008; Raver and Nishii, 2010; Nielsen and Einarsen, 2012). Thus, further understanding the impact of discrimination on workplace experiences could have measurable consequences for organizations above and beyond a desire for fairness and equality (Bobo, 2001).

A variety of legal protections, such as the Civil Rights Act (1964, 1991), have helped to curb overt expressions of discrimination in the workplace (Dipboye and Colella, 2004; Aiken et al., 2013). Yet, it is clear that discrimination still lingers in less overt forms. Researchers distinguish between two forms of workplace discrimination—formal (i.e., overt) and interpersonal (i.e., subtle) discrimination (Hebl et al., 2002, 2015). Formal discrimination, on the one hand, can be defined as “discrimination in hiring, promotions, access, and resource distribution…that in many states is illegal… [and against which] there are often organizational laws, company policies, or social norms” (Hebl et al., 2002, p. 816). Such discrimination is hostile, overt, and often based on the belief that members of a stigmatized group are inferior to their non-stigmatized peers (Cortina, 2008). Interpersonal discrimination, on the other hand, is characterized more often by negative “non-verbal, paraverbal, and even some verbal behaviors that occur in social interactions” (Hebl et al., 2002, p. 819). These behaviors are typically enacted toward members of a stigmatized group; however, such behaviors are more ambiguous in intent and often difficult to prevent (Rowe, 1990; Cortina, 2008; Jones et al., 2013). Unlike formal discrimination, interpersonal discrimination is not legislated, partly because it can be difficult to categorize. Interpersonal discrimination, also discussed under the label of incivility (e.g., Cortina et al., 2011; Miner et al., 2012), may be dismissed as rudeness; however, like formal manifestations of discrimination, interpersonal discrimination reflects targeted (and often repeated) behaviors directed toward a person based on their membership in a stigmatized group.

Research suggests that subtle forms of discrimination are at least as important in impacting a target’s behavior as formal discrimination, if not more so (Jones et al., 2013). Although subtle discrimination colloquially has been perceived as less injurious (see Valian, 1999), empirical research suggests that this is not necessarily the case. For instance, in an experimental study examining the impact of both formal and interpersonal discrimination, Singletary (2009) found that participants asked to perform workplace tasks who were exposed to interpersonal discrimination demonstrated deficits in performance on an in-basket exercise and experienced significant attentional resource depletion, whether or not formal discrimination was present. Results showed that attentional resource depletion mediated the relation between interpersonal discrimination and performance; that is, the attributional ambiguity of interpersonal discrimination seems to lead participants to exert more cognitive effort while interpreting negative interpersonal behaviors. Thus, the subtlety of interpersonal discrimination (see Crocker et al., 1998), coupled with the simple rudeness inherent to interpersonal discrimination, negatively impacts job-related outcomes far more than does formal discrimination, which can be more easily identified and attributed wholly to prejudiced others.

Despite preliminary research demonstrating that interpersonal discrimination negatively affects employees’ performance and psychological well-being, more work is needed to understand the impact of interpersonal discrimination in the workplace. One reason that such research still is relatively sparse is that diversity initiatives in the workplace often are targeted at reducing overt, rather than subtle, discriminatory behaviors (Shih et al., 2013). Understanding the role of interpersonal discrimination at work is vital for mitigating the potential impact of discrimination on employee outcomes. Thus, we next detail several ways in which interpersonal discrimination might impact stress, health, and work outcomes.

### The Impact of Discrimination at Work

Broadly, perceived discrimination has been linked to decrements in mental and physical well-being (e.g., Pascoe and Richman, 2009). In a recent meta-analysis, Jones et al. (2013) found a meaningful correlation between interpersonal discrimination and psychological distress (*r_c_* = 0.28). Additional research suggests a causal relation—perceptions of discrimination are linked to negative mental health outcomes several years after the initial incidence of discrimination (Pavalko et al., 2003). Similarly, employees who frequently encounter incivilities from others in their organization display greater feelings of psychological distress, including higher rates of depression and anxiety (Cortina et al., 2001).

The impact of discrimination on stress has implications for work outcomes and physical well-being. A robust body of research suggests that the experience of stress can lead to substantial negative physical outcomes (e.g., DeLongis et al., 1988; Pascoe and Richman, 2009). Pascoe and Richman (2009) state that, although the relation between discrimination and negative mental health outcomes is strongly supported, relations between discrimination and physical health outcomes are less frequently examined. For instance, Goldman et al. (2006) observed that gender-based discrimination related to the onset of physical ailments in women. Supporting this, meta-analytic evidence has shown that discrimination correlates with increased incidents of physical health issues (*r_c_* = 0.16; Jones et al., 2013).

In considering the links between discrimination and health, some propose that discrimination, like more overt forms of workplace aggression, is a type of stressor (Zapf and Einarsen, 2005; Hansen et al., 2006; Hauge et al., 2010). Specifically, interpersonal discrimination induces substantial attributional ambiguity, is often uncontrollable, and can occur without warning—with increased health maladies as a potential negative outcome of the experience of discrimination-related stress (Gee et al., 2007; Williams and Mohammed, 2009). Specifically, interpersonal discrimination may function as a stressor that makes employees more vulnerable to psychological and physical health maladies (Gee et al., 2007). Additionally, a person’s mental health status may act as a mediator between the experience of discrimination and physical health outcomes. In a study of the impact of incivility on mental and physical health, Lim et al. (2008) found that employees experiencing frequent interpersonal discrimination were more likely to experience increased psychological distress (i.e., depression and anxiety). Mental health status, in turn, was negatively linked to quality of physical health. Moreover, the experience of discrimination had immediate negative impacts on mental health, while the continued strain of discrimination also produced physical ailments over time (Lim et al., 2008).

In addition to physical and mental health declines, the experience of discrimination, harassment, bullying, and other forms of workplace aggression has been linked to myriad organizational outcomes (e.g., Raver and Nishii, 2010; Nielsen and Einarsen, 2012). For instance, women who perceive sexual harassment at work demonstrate greater organizational withdrawal and lower job satisfaction, organizational commitment, and productivity (Willness et al., 2007). Similarly, perceived discrimination among ethnically diverse employees has been linked to lower organizational commitment and job satisfaction, and to decreased engagement in organizational citizenship behaviors (OCBs: Ensher et al., 2001), also known as contextual performance (see Organ, 1997; Motowidlo, 2000). Interpersonal discrimination, in particular, has been linked to increased turnover intentions (Lim et al., 2008; Cortina et al., 2011), lower organizational commitment (Schat and Frone, 2011), and lower job satisfaction (Cortina et al., 2001; Deitch et al., 2003; Miner et al., 2012). More generally, exposure to workplace aggression has been linked to poor task and discretionary performance (Schat and Frone, 2011). In response to discrimination, employees may decrease their OCBs (Ensher et al., 2001; Singletary, 2009) and increase deviant workplace behaviors (Kickul et al., 2001). In sum, findings link interpersonal discrimination to a wide variety of job outcomes and highlight the many negative consequences for those who experience discrimination and their organizations.

### Current Study

In the present study, we focus on the consequences of perceived interpersonal discrimination on stress, health, and performance. In so doing, we contribute to the literature in three ways: first, our study uses a sample of academicians in STEM fields. Although, research has demonstrated the deleterious effects of overt forms of workplace aggression on educators and academicians health and well-being (e.g., Björkqvist et al., 1994; Fox and Stallworth, 2010), more research is needed to understand the role of interpersonal discrimination in the academic context, particularly with respect to performance outcomes. Diverse academicians in STEM fields face careers with more social isolation, slower promotion through the academic ranks, and fewer mentors than their majority peers (e.g., Settles, 2014). Often referred to as the “leaky pipeline,” many such individuals leave their STEM training and careers (Etzkowitz et al., 2000; Metcalf, 2010), and experiences of discrimination may be a particularly salient driver of why some individuals choose to leave STEM fields (Riﬄe et al., 2013). By understanding how discrimination manifests in health and performance outcomes, we might better mitigate the negative impact of these experiences.

Second, the present study extends the work of researchers such as Lim et al. (2008) and Jones et al. (2013) by examining the impact of interpersonal discrimination on job-related performance outcomes in addition to physical and psychological health. Although, psychological and physical well-being are critically important outcomes, additional clarity regarding relations between discrimination, particularly interpersonal discrimination, and performance outcomes is greatly needed. Thus, by extending the consequences of interpersonal discrimination examined to include those of individual and organizational relevance we hope to gain a more nuanced understanding of effects of discrimination at work. Specifically, we sought to examine stress as a potential mediator between experiences of interpersonal discrimination and physical health, and physical health as a potential mediator between stress and performance (Deitch et al., 2003; Singletary, 2009; Jones et al., 2013).

We examined performance in STEM academicians by capturing indicators of performance from both the task and contextual domains (Borman and Motowidlo, 1993). First, we elected to use an objective measure of performance in STEM academicians: namely, the participants’ publishing productivity combined with citation count (i.e., h-index), collected 3 years after the completion of the initial survey. Second, in addition to this more objective measure of task performance, we also measured discretionary workplace behavior in the form of OCBs (Smith et al., 1983). As demonstrated by Singletary (2009), interpersonal discrimination negatively impacts performance and reduces intentions to engage in future helping behaviors. We therefore examined participants’ engagement in OCBs as they relate to the experience of interpersonal discrimination. Ensher et al. (2001) found that 300 non-White employees ratings of perceived discrimination from coworkers, supervisors, and the organization were related to decreased OCBs, as well as decreased job satisfaction and organizational commitment. We note that Ensher et al. (2001) did not examine the mechanism linking perceived discrimination to OCBs. Thus, the current study extends past research by exploring potential contributors to feelings of discrimination and the translation of those feelings into behaviors.

Third, and finally, a large body of research supports the mitigating role of social support for reducing stress perceptions (e.g., Johnson and Hall, 1988; Spector, 1998; Van der Doef and Maes, 1999). Within an organizational context, social support has been linked to less reported stress, better interpersonal relationships, less turnover, and higher job satisfaction (Viswesvaran et al., 1999). For instance, Miner et al. (2012) found that social support lessened the impacts of perceived incivility on stress and job satisfaction in a sample of property management employees, and similarly, that social support mitigated the effects of perceived gender-based incivility on depression and general life satisfaction in a sample of college students. Using the guiding framework of the “buffering hypothesis” (LaRocco et al., 1980; Karasek et al., 1982; Johnson and Hall, 1988), we expect that supervisor support will buffer STEM academicians against the negative effects of discrimination on stress. Specifically, social support may act as a buffer between the experience of a stressful event and the appraisal of that event as stressful (Dignam and West, 1988). Thus, we include supervisor social support as a potential moderator of the relation between perceived interpersonal discrimination and stress.

In line with previous research on the importance of social support in the workplace as a buffer against stress (e.g., Dignam and West, 1988; Miner et al., 2012) and guided by past research on the impact of discrimination on stress (e.g., Pascoe and Richman, 2009; Jones et al., 2013), we hypothesize that interpersonal discrimination will be positively related to stress, and that supervisor support will moderate this relation:

Hypothesis 1a: Interpersonal discrimination will be positively related to stress.

Hypothesis 1b: Supervisor support will moderate the relation between interpersonal discrimination and stress.

As noted, the impact of interpersonal discrimination on psychological well-being is well-documented (e.g., Jones et al., 2013); yet associations between such interpersonal discrimination and physical health outcomes are under researched (Pascoe and Richman, 2009). Keeping with recent research on the impact of discrimination-related stress on physical health outcomes (e.g., Goldman et al., 2006; Lim et al., 2008), we predict that stress will be related positively to incidence of health maladies, and that stress will mediate the relation between interpersonal discrimination and health maladies:

Hypothesis 2a: Stress will be positively related to incidence of health maladies.

Hypothesis 2b: Stress will mediate the relation between interpersonal discrimination and health maladies.

Beyond mental and physical health outcomes, stress resulting from perceptions of interpersonal discrimination has been linked to performance decrements in the workplace (e.g., Ensher et al., 2001; Jones et al., 2013). Extending from this research, we predict that stress will be related negatively to STEM academicians’ academic productivity and OCBs, and that stress will mediate the relations between interpersonal discrimination and these performance outcomes:

Hypothesis 3a: Stress will be negatively related to academic productivity and OCBs.

Hypothesis 3b: Stress will mediate the relation between interpersonal discrimination and academic productivity and OCBs.

One mechanism through which discrimination-related stress might negatively influence performance outcomes is declines in physical well-being. To that end, we posit that the incidence of health maladies will relate negatively to STEM academicians academic productivity and OCBs, and that incidence of health maladies will mediate the relation between stress and performance.

Hypothesis 4a: Incidence of health maladies will be negatively related to academic productivity and OCBs.

Hypothesis 4b: Health maladies will mediate the relation between stress and academic productivity and OCBs.

**FIGURE 1:**
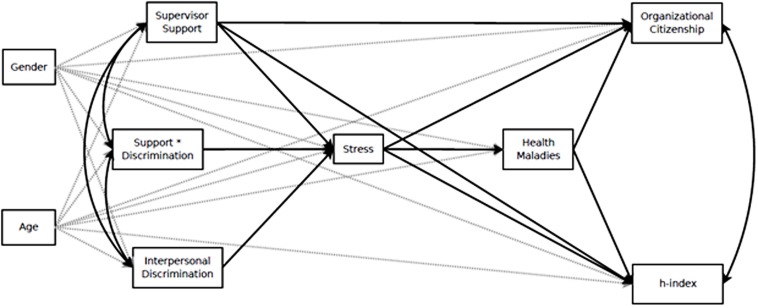
**Hypothesized path model of the relations between interpersonal discrimination, health, and performance in STEM academicians**.

**Figure 1** depicts the hypothesized path model for the associations between interpersonal discrimination, supervisor support, and participants’ health and performance outcomes, through the effects of discrimination on stress.

## Materials and Methods

### Participants

Participants were recruited as part of a larger study through a National Science Foundation ADVANCE Grant Initiative via targeted email solicitations and online via social media participation requests targeting early career academics. Two hundred twenty STEM academicians from universities across the U.S. completed an online study examining their workplace experiences. Of these, 136 identified as female (61.8%), 79 identified as male (35.9%), and 5 did not specify a gender (2.3%). Participants listed their race as White (73.2%), Asian (15.0%), Black (3.6%), Hispanic (2.7%), Multiracial (2.7%), or did not specify (2.7%). Participants were between 24 and 52 years-old (*M* = 32 years; *SD* = 4.88 years) and included graduate students (30.0%), postdoctoral researchers (27.3%), and faculty members (42.8%), having held their current position from 1 month to 8.3 years (*M* = 1.82; *SD* = 1.77). Ten participants did not have available data for key outcomes measures and were removed from the analysis, resulting in a final sample of 210 STEM academicians.

### Measures

#### Interpersonal Discrimination

Interpersonal discrimination was measured with Cortina et al.’s (2001) seven-item incivility scale. Participants rated the extent to which they perceived the experience of interpersonal discrimination in the workplace on items such as “ignored or excluded you from professional comradery” and “made demeaning or derogatory remarks about you” on a five-point Likert scale (1 = *never* to 5 = *most of the time*).

#### Supervisor Support

Supervisor support was measured using eight items adapted from the Job Content Questionnaire (Karasek et al., 1998). All items reflected the instrumental and emotional support participants perceived from their supervisors and included items such as “My supervisor is helpful at getting the job done” and “My supervisor takes an active interest in my career development.” Participants rated items on a nine-point Likert scale (1 = *not at all true of me*, 9 = *very true of me*).

#### Health Maladies

Participants rated the frequency with which they experienced 14 common health maladies including: (1) headaches; (2) a stomach ache or upset stomach; (3) cold sweats, particularly at night from stress; (4) feeling physically weak; (5) feeling very tired out, just exhausted; (6) feeling sick, just not feeling 100%; (7) waking up feeling tired; (8) skin problems, such as itching, acne, or scab picking; (9) dizziness; (10) chest pains; (11) aches, pains, or soreness in muscles or joints; (12) painful or frequent urination; (13) sore throat or a cough; and (14) hot flashes. Items were rated on a four-point Likert scale (1 = *never*, 2 = *just a few times*, 3 = *about once a week*, and 4 = *almost every day*), and are similar to those used in existing research on the effects of discrimination and stress on physical health outcomes (e.g., Cohen and Hoberman, 1983; Denton et al., 2014).

#### Stress

Stress was measured using Cohen et al. (1983) 10-item measure of perceived stress. Participants indicated the extent to which they had felt stress in the past month on items such as: “In the last month, how often have you felt nervous or ‘stressed’?” and “In the last month, how often have you been upset because of something that happened unexpectedly?” Participants rated items on a seven-point Likert scale (1 = *never*, 7 = *always*).

#### Organizational Citizenship Behaviors

Participants rated their perceived levels of organizational citizenship behaviors on a 34-item measure by Organ (1988). Participants indicated the extent to which they agreed with items such as “I represent the organization favorably to outsiders” and “I rarely waste time while at work” on a seven-point Likert scale (1 = *strongly disagree*, 7 = *strongly agree*).

#### h-index

Participants’ objective performance in academia was measured 3 years after the original survey administration using participants’ h-index, retrieved from Scopus^1^. The h-index is a measure of academic productivity and impact, and is based on the number of citations that the researchers’ most cited papers receive (Hirsch, 2005). For the current sample, h-indices ranged from 0 to 36 and demonstrated slight positive skew (2.55) and moderate kurtosis (9.34). We therefore used a natural log transformation of this variable in our path analysis. The inclusion of this lagged measure allows us to examine any potential extended effects of discrimination on STEM academicians’ objective performance outcomes.

## Results

Descriptive statistics and correlations are presented in **Table 1**. As shown in the table, there were small, positive correlations between age and OCBs and, not surprisingly h-index, suggesting that older STEM academicians demonstrated higher levels of these outcomes. Moreover, there was a small, negative association between gender and interpersonal discrimination, such that women experienced higher levels of interpersonal discrimination. Interestingly, there was a moderate negative association between gender and OCBs, such that women reported performing more OCBs than men, suggesting that women might engage in greater amounts of service in academic compared to men. Interpersonal discrimination demonstrated small-to-moderate positive correlations with participants’ perceived stress and incidence of health maladies. Moreover, supervisor support was related negatively to these outcomes, yet was related positively to participants’ engagement in OCBs. In addition, health maladies were correlated positively with participants’ stress perceptions. Notably, stress perceptions also were related negatively to participants’ engagement in OCBs.

**Table 1:** Descriptive statistics and correlations for raw study measures.

	*Mean*	*SD*	1	2	3	4	5	6	7
(1) Gender^1^	0.35	–	–						
(2) Age	32.01	4.88	–0.07	–					
(3) Interpersonal discrimination	2.04	0.94	–0.14	0.01	*0.93*				
(4) Supervisor support	6.63	1.96	–0.02	–0.10	**–0.35**	*0.94*			
(5) Stress	3.16	0.79	0.01	0.12	**0.29**	**–0.29**	*0.87*		
(6) Health maladies	1.66	0.36	–0.04	–0.03	**0.22**	**–0.18**	**0.34**	*0.81*	
(7) OCB	4.95	0.67	–**0.30**	0.08	**–0.15**	**0.26**	–**0.22**	–0.12	*0.92*
(8) h-index	5.58	5.08	–0.14	**0.16**	0.11	0.01	–0.07	0.13	0.14

*Listwise N = 182. ^1^Coded 0 = female, 1 = male. Correlations in **bold** are statistically significant, p < 0.05. Alphas are on the diagonal in italics.*

**FIGURE 2:**
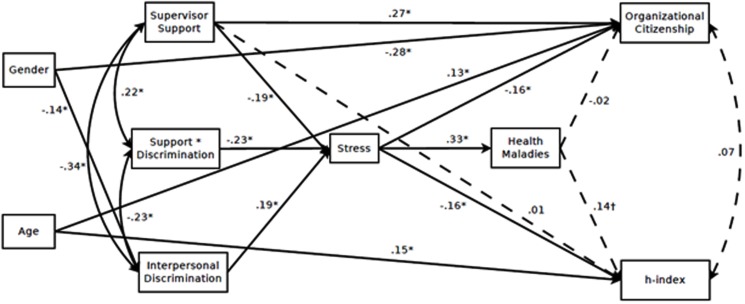
***N* = 210.** Standardized coefficients for path model. Dashed lines are statistically non-significant. Non-significant paths from covariates are not depicted. ^∗^*p* < 0.05, ^†^*p* = 0.06.

Our hypothesized path model was tested in Mplus v.7.2 (Muthén and Muthén, Muthén and Muthén) using maximum likelihood estimation. Analyses were conducted at the level of the scale scores, and interpersonal discrimination, supervisor support, stress, and health maladies were grand mean centered to foster interpretability. Gender and age were entered as covariates in the model given known differences in the experiences of men and women within STEM academia that might confound the effects of interpersonal discrimination (e.g., Riﬄe et al., 2013)^2^, and that age should meaningfully and positively relate to number of publications and citations. Missing data were estimated using full information maximum likelihood (FIML) procedures. Overall, this model demonstrated excellent close and exact fit to the data (χ^2^(7) = 7.41, *p* = 0.39; CFI = 0.997; RMSEA = 0.017 [0.000, 0.088]; SRMR = 0.023). Standardized coefficients are presented in **Figure 2**.

**FIGURE 3:**
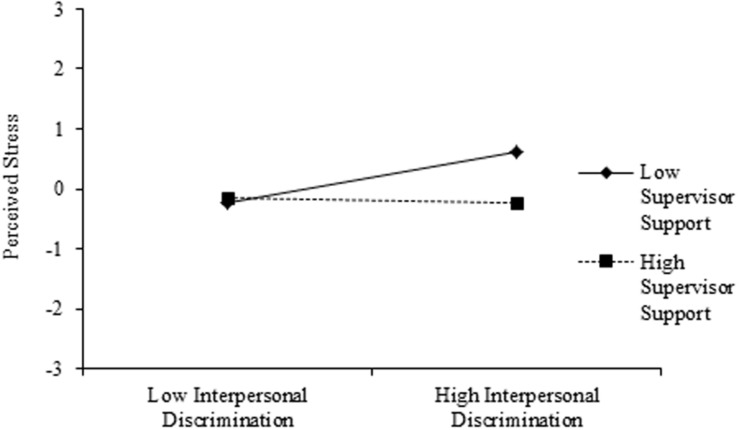
**Standardized interaction between perceived interpersonal discrimination and supervisor support predicting perceived stress.** Parameters obtained from path model (**Figure 2**).

Hypothesis 1 predicted that perceived interpersonal discrimination would positively predict stress (H1a) and that supervisor support would moderate this relation (H1b). Consistent with our expectations, there was a significant positive relation between interpersonal discrimination and stress (β = 0.19, *SE* = 0.07, *p* < 0.01). Specifically, STEM academicians who experienced more interpersonal discrimination in their work environment also reported greater levels of stress. Moreover, this relation was qualified by an interaction between supervisor support and interpersonal discrimination (β = –0.23, *SE* = 0.06, *p* < 0.001), such that supervisor support buffered participants from experiencing stress as a result of perceived interpersonal discrimination (see **Figure 3**). Thus, Hypothesis 1 was supported.

Hypothesis 2 predicted that stress would be related to health maladies (H2a) and that stress would mediate the relation between perceived interpersonal discrimination and health maladies (H2b). This relation was supported, such that there was a moderate positive relation between perceived stress and the incidence of health maladies (β = 0.33, *SE* = 0.06, *p* < 0.001). In addition, stress mediated the relation between interpersonal discrimination and health maladies (*I* = 0.06, *SE* = 0.03, *p* = 0.04, 95%CI = 0.00, 0.13)^3^. Thus, Hypothesis 2 was supported. Though not hypothesized explicitly, stress also was found to mediate the relation between supervisor support and the incidence of health maladies (*I* = –0.06, *SE* = 0.03, *p* = 0.01, 95%CI = –0.12, –0.01).

Hypothesis 3 predicted that stress would be negatively related to performance (H3a) and that stress would mediate the relation between perceived interpersonal discrimination and performance (H3b). As expected, stress was negatively related to STEM academicians’ OCBs (β = –0.16, *SE* = 0.07, *p* = 0.02) and h-index (β = –0.16, *SE* = 0.08, *p* = 0.03), such that academicians who reported more stress indicated lower levels of OCBs at the time of reported perceived interpersonal discrimination, and had lower h-indices 3 years later. Thus, Hypothesis 3a was supported. However, stress did not mediate the relations between perceived interpersonal discrimination and OCBs (*I* = –0.03, *SE* = 0.02, *p* = 0.09, 95%CI = –0.07, 0.01) or between perceived interpersonal discrimination and objective performance (*I* = –0.03, *SE* = 0.02, *p* = 0.10, 95%CI = –0.07, 0.01). Thus, Hypothesis 3b was not supported.^4^

Hypothesis 4 predicted that incidence of health maladies would negatively relate to performance (H4a) and that health maladies would mediate the relation between stress and performance (H4b). Contrary to our expectations, health maladies predicted neither OCBs (β = –0.02, *SE* = 0.07, *p* = 0.75) nor h-index (β = 0.14, *SE* = 0.08, *p* = 0.06), thus failing the necessary conditions for mediation (e.g., Baron and Kenny, 1986). Additionally, the relation between health maladies and objective performance was opposite of the predicted direction. As such, performance in STEM academicians appears to be less dependent on the physical health, but rather on mental health as measured by stress. Thus, Hypothesis 4 was not supported, but did offer insights into the impact of stress on health and performance outcomes in academia.

## Discussion

The present study extends important research examining the effects of interpersonal discrimination on physical and psychological well-being and performance (e.g., Cortina et al., 2001; Lim et al., 2008; Miner et al., 2012; Jones et al., 2013). Findings indicate that interpersonal discrimination impacts STEM academicians’ stress perceptions and, critically, that stress negatively impacts STEM academicians’ OCBs, physical health, and academic productivity measured several years later. Examining outcomes associated with interpersonal discrimination is important given that it has not been the focus of much prior organizational research (Jones et al., 2013). Although, interpersonal discrimination might be perceived as being nothing more than a series of unimportant complaints, molehills, and minor transgressions that have little impact (Valian, 1999), the findings of the current research offer very different conclusions. Namely, the experience of interpersonal discrimination has important negative implications for STEM academicians’ physical and psychological health and well-being. Understanding the implications of interpersonal discrimination within STEM fields offers one potential explanation of the consequences of lingering inequities experienced by non-majority members in these fields (Tolbert, 1998; Metcalf, 2010).

In support of our model, perceptions of interpersonal discrimination lead to greater levels of stress. This effect was moderated by supervisor support such that those who had greater levels of support were buffered from the effects of interpersonal discrimination, consistent with the findings of Griffith and Hebl (2002; see also, Miner et al., 2012). This is an important finding for STEM academicians, namely that supervisor support can make a difference while one is establishing or even after one has established a career. Furthermore, we found that stress leads to greater incidence of health maladies, and results suggest that stress mediates the relation between interpersonal discrimination and health maladies. Thus, even before evaluating performance for STEM academicians, we found meaningful mental and physical health outcomes of interpersonal discrimination, which were, in part, buffered by perceived supervisor support.

Contrary to our expectations, our model did not support the prediction that incidence of health maladies would negatively affect performance. Instead, negative performance outcomes were primarily driven by psychological stress. This suggests that mental health and stress have a greater impact on STEM academicians’ performance than do physical maladies. Rather than considering psychological and physiological outcomes to be equal antecedents of performance, instead we should consider psychological distress to be a direct antecedent of physiological health and of performance outcomes. Also contrary to our expectations, stress did not appear to mediate relations between perceptions of interpersonal discrimination and STEM academicians’ performance outcomes. Note, however, that meaningful relations were observed between interpersonal discrimination and stress, and between stress and our performance outcomes, in turn. This suggests the potential for implied mediation; however, the indirect effects observed in the present study did not reach (though approached) the level of statistical significance—a potential result of our modest sample size. Future, large-sample research implementing robust experimental designs is needed to examine the extent to which stress mediates the relation between the experience of interpersonal discrimination and performance in academia, given its substantial implications for understanding the underrepresentation of demographically diverse groups in STEM fields.

As a whole, the present research suggests that experiencing interpersonal discrimination in the workplace leads to greater levels of stress, which in turn leads to decrements in performance. Moreover, stress mediates the relation between interpersonal discrimination and decrements in physical health. On a more encouraging note, supervisor support was found to be a significant moderator on the effect of interpersonal discrimination on stress, lessening the effects of interpersonal discrimination on STEM academicians’ level of stress, and thus its decrements to performance and health. This finding is consistent with research showing that those who are stigmatized benefit tremendously by having supportive colleagues (Griffith and Hebl, 2002; Miner et al., 2012). Experiencing persistent and pernicious interpersonal discrimination may drain emotional and cognitive resources resulting from increased cognitive load due to attributional ambiguity (Crocker et al., 1991), similar to processes observed for emotion regulation in the workplace (Trougakos et al., 2015). Specifically, the experience of interpersonal discrimination depletes employees’ emotional resources such that employees are less likely to engage in altruistic behaviors (cf. Ensher et al., 2001; Singletary, 2009), and are more likely to engage in reciprocal exchanges of incivility (Andersson and Pearson, 1999) and counterproductive work behaviors (Zhou et al., 2014).

### Considerations and Future Directions

The present research focused on the experience of early career STEM academicians. Although, concerns regarding interpersonal discrimination resulting from demographic status are, and should be, ubiquitous across academic disciplines, discrimination toward individuals of minority status in STEM fields is of paramount importance as such discrimination may contribute to the “leaky pipeline.” Note, however, that not all STEM fields are created equal with respect to differences in minority membership (Ceci et al., 2014; Su and Rounds, 2015). Rather, recent research has highlighted the need to distinguish mathematically intensive STEM fields (e.g., engineering, mathematics, computer science, and the physical sciences), in which men are typically overrepresented, from less mathematically oriented STEM fields (e.g., life sciences, psychology, and the social sciences; Halpern, 2014). As such, future research should consider this distinction to understand more fully the contexts in which discrimination occurs in STEM fields. More broadly, although academia provides an important context for understanding the effects of interpersonal discrimination, it is important to assess whether these findings generalize to STEM practitioners outside of academia.

Due to the well-documented inequities in STEM fields between genders (e.g., Halpern et al., 2007; Riﬄe et al., 2013; Ceci et al., 2014), we controlled for participant gender in the present study. Note, however, that the use of a multiple-groups design would also be appropriate for testing our model within different demographic groups; however, the present study was limited in its ability to test for the moderating effects of demographic characteristics, such as gender, given the size and composition of our sample. Future research should extend to other factors that affect experiences of discrimination including demographic differences other than gender (e.g., race, religion, and disability status; see Goldman et al., 2006) and differences in cultural values at the organizational and cultural levels (e.g., discrimination tolerance; Arenas et al., 2015; Giorgi et al., 2015). Similarly, the use of multiple-groups designs would allow researchers to determine boundary conditions with respect to mathematically intensive vs. less mathematically intensive STEM fields.

Although, it is important to understand the effects of discrimination on employee outcomes, it is vital that organizational researchers discover and understand remediation strategies to reduce the incidence of such discrimination in the workplace. Findings of the present study reinforce the role of supervisor support as a buffer against interpersonal discrimination in STEM fields. Organizations, however, should not merely rely on formal supervisors or academic mentors to assuage the impact of interpersonal discrimination; rather, organizations should proactively pursue strategies to mitigate, if not prevent, such subtle forms of discrimination before they occur. For instance, Leiter et al. (2011, 2012; see also Osatuke et al., 2009) have shown that incivility interventions in health care occupations can be used to decrease the incidence of interpersonal discrimination in the workplace, to foster employees’ job-related attitudes and psychological well-being, and to reduce the incidence of turnover and absenteeism. As such, future research should place greater emphasis on understanding remediation strategies that can be implemented by individuals, bystanders, and institutions to end interpersonal discrimination.

A final consideration for the present study is how success in early career STEM academicians is conceived. Academicians’ performance is multidimensional, as evidenced by the growing body of research on scholarly impact (Aguinis et al., 2012, 2014). Although, scholarly impact is frequently captured by obtaining citation counts (e.g., Podsakoff et al., 2008), there are other ways in which impact might be operationalized beyond citations in academic outlets (e.g., number of press releases, references on non-.edu websites; see Aguinis et al., 2012, 2014). Moreover, citation metrics such as h-index have been criticized as a measure of scholarly impact (e.g., Costas and Bordons, 2007; Waltman and Van Eck, 2012; Waltman et al., 2012; but see, e.g., Ruscio et al., 2012). Although the present study represents a positive step toward understanding the multidimensional nature of scholarly impact in our consideration of academicians’ objective productivity (i.e., h-index) and engagement in OCBs, our performance outcomes are limited to academicians’ behavior within the Academy. Thus, future research should consider the influence of interpersonal discrimination and physiological and psychological health on academic success using a pluralist conception of scholarly impact.

## Conclusion

Understanding the negative impact of interpersonal discrimination on the health and productivity of workers adds additional support to the need to mitigate and eliminate discrimination in all forms. Interpersonal discrimination increases employees’ stress levels and the experience of physical health maladies, and the stress arising from such discrimination decreases altruistic behavior at work, in turn. Moreover, these experiences linger. Objective performance decrements arising from stress due to perceived interpersonal discrimination can be detected among STEM academicians several years after they report it. Social support is one important way in which others can help employees experiencing interpersonal discrimination, but such interventions treat the symptoms rather than the disease. Instead, understanding the implications of interpersonal discrimination brings us one step closer to eliminating its causes.

## Author Contributions

KO: collected data, assisted with statistical models, and co-wrote the paper; SM: ran statistical models and co-wrote the paper; MH: assisted in data collection, edited the paper, oversaw the project, and contributed financial backing; JR: contributed financial backing and oversaw the writing of the paper.

## Conflict of Interest Statement

The authors declare that the research was conducted in the absence of any commercial or financial relationships that could be construed as a potential conflict of interest. The reviewer KM and handling Editor declared their shared affiliation, and the handling Editor states that the process nevertheless met the standards of a fair and objective review.

## Footnotes

1 www.scopus.com

2 As a sensitivity analysis, we also tested our model for women only (*n* = 132). The general pattern of findings was largely similar to those reported; however, statistical power was noticeably reduced. We therefore retained the full sample of both men and women for all analyses, and elected to include gender as a covariate. We thank a reviewer for this suggestion.

3 We use Bollen’s (1987) designation, *I*, to refer to the specific indirect effects, along with their associated *p*-values and bias-corrected bootstrapped confidence intervals (see Shrout and Bolger, 2002; Cheung and Lau, 2008).

4 An alternative model that included direct paths from interpersonal discrimination to incidence of health maladies, OCBs and h-index was tested. This model demonstrated a non-significant change in fit [Δχ^2^(3) = 3.96, *p* > 0.05], and all direct paths from interpersonal discrimination to these outcomes were statistically non-significant (*p* > 0.05). We therefore retained our initial model.
